# Effectiveness of a therapeutic program with chin tuck against resistance exercises compared to the chin-down maneuver in Parkinson's disease: a randomized controlled pilot trial

**DOI:** 10.1590/2317-1782/e20250191en

**Published:** 2026-06-15

**Authors:** Ramon Cipriano Pacheco de Araújo, Karinna Veríssimo Meira Taveira, Juliana Fernandes Godoy, Hipólito Magalhães

**Affiliations:** 1 Programa de Pós-graduação em Fonoaudiologia, Universidade Federal do Rio Grande do Norte – UFRN - Natal (RN), Brasil.; 2 Núcleo de Estudos Avançados em Revisão Sistemática e Meta-análise – NARSM, Programa de Pós-graduação em Fonoaudiologia, Universidade Federal do Rio Grande do Norte – UFRN - Natal (RN), Brasil.

**Keywords:** Deglutition Disorders, Deglutition, Parkinson's Disease, Exercise, Swallowing Rehabilitation

## Abstract

**Purpose:**

Investigate the effectiveness of a therapeutic program with chin tuck against resistance (CTAR) exercises, compared to the chin-down (CD) maneuver, on signs suggestive of dysphagia, maximum isometric tongue pressure, and therapeutic efficacy in individuals with Parkinson’s disease (PD).

**Methods:**

26 individuals with primary PD were randomly assigned to two intervention groups for four weeks (one session per week). Group A performed the CTAR exercises (n = 13), and Group B performed the CD postural maneuver with swallowing training with different food consistencies (n = 13). The outcomes assessed were signs suggestive of dysphagia using the Northwestern Dysphagia Patient Check Sheet (NDPCS), maximum isometric tongue pressure (MIP), the Eating Assessment Tool (EAT-10) to monitor therapeutic efficacy, and peak cough flow, before and after the intervention period.

**Results:**

The CTAR group showed a significant improvement in items related to the NDPCS, MIP, and EAT-10 compared to the CD group (p < 0.05). There was no significant difference in peak cough flow in either group.

**Conclusion:**

PD patients who underwent an intervention program with CTAR exercises showed an improvement in signs suggestive of dysphagia and an increase in maximum isometric tongue pressure, compared to those who underwent the therapeutic program with the CD postural maneuver.

## INTRODUCTION

Parkinson's Disease (PD) is the second most common neurodegenerative disease in the population, with an increasing prevalence with advancing age^(1)^. This debilitating condition is known to affect both the central and peripheral nervous systems, with the characteristic histopathological presence of alpha-synuclein aggregates^(2)^. Although it involves degeneration of the nigrostriatal dopaminergic pathway, PD also affects other neural pathways, resulting in complex functional deficits such as dysarthria, sialorrhea, and oropharyngeal dysphagia^(3)^.

Swallowing disorders are associated with advanced stages of the disease, but can be present from the earliest stages, even with mild alterations^(3,4)^. The prevalence of oropharyngeal dysphagia in PD varies between cases, depending on the type of assessment carried out^(5,6)^. In clinical speech therapy practice, specifically in less developed countries or regions, instrumental assessment is not always feasible due to the substantial costs^(7)^. In this context, the application of reliable clinical assessment protocols is essential for defining the diagnostic hypothesis and monitoring dysphagia. This makes it possible to understand the alterations in the biomechanics of swallowing and to draw up an appropriate therapeutic plan^(8,9)^.

In PD, the reduced efficiency of lower airway protection mechanisms increases the risk of aspiration during swallowing^(10)^. This impairment can be attributed to the reduction in pharyngeal response and the reduction in expiratory cough flow, which is responsible for eliminating aspirated waste^(10)^. As a result, the components responsible for swallowing safety become less effective in PD patients^(5,10)^.

Among the therapeutic approaches for dysphagia in PD, the use of compensatory strategies is widely used^(11)^. The chin-down postural maneuver (CD) is one of the most investigated interventions for reducing aspiration episodes in this population^(11-13)^. Studies have shown that, when combined with the ingestion of thickened liquids, this maneuver reduces pharyngeal residues after swallowing and, consequently, aspiration^(12,13)^. As this is a compensatory technique, the changes in the biomechanics of swallowing are transitory. This prevents long-term training, which represents a significant limitation when faced with the progression of the disease^(11)^.

From this perspective, a promising exercise in dysphagia rehabilitation is the chin tuck against resistance (CTAR)^(14)^. Evidence shows increased activation of the suprahyoid muscles, with isotonic and isometric components, in different pathophysiological changes in swallowing^(14,15)^. To date, we have found no published data on its effects in PD patients. Although the two interventions present different proposals about the biomechanics of swallowing, both are used in clinical practice to improve the clinical signs of dysphagia^(11-15)^.

The comparison between different interventions related to swallowing has already been explored in the literature and can contribute to new therapeutic perspectives and the direction of future research, especially in PD^(13)^. To verify differences between two interventions applied in clinical practice, we hypothesized that a program based on CTAR exercises is more effective in improving clinical signs of dysphagia than the CD maneuver. This study aimed to investigate the effectiveness of a therapeutic program with CTAR exercises, compared to the CD maneuver, on signs suggestive of dysphagia, maximum isometric tongue pressure, and therapeutic efficacy in individuals with PD.

## METHODS

This is a pilot study in the form of a randomized clinical trial, registered with the Brazilian Registry of Clinical Trials (number U1111-1299-7361), and followed the methodology proposed by CONSORT^(16)^ using the PICO (Patient, Intervention, Comparison and Outcome) strategy, based on the question: Would individuals with PD who underwent CTAR exercises, when compared to those who underwent the CD maneuver, show improvements in signs suggestive of dysphagia, maximum isometric tongue pressure, therapeutic efficacy and cough function?

The study was conducted at the movement disorders outpatient clinic of the Hospital Universitario Onofre Lopes between August 2023 and October 2024. The study was approved by the Research Ethics Committee of the Hospital Universitario Onofre Lopes (number: 6,270,789). Written consent was obtained from all participants prior to the research procedures

### Participants

The inclusion criteria were: age over 18, a clinical diagnosis of Parkinson's disease, being able to understand and follow the commands correctly, having the motor skills to perform both exercises correctly, and having identified complaints of oropharyngeal dysphagia using the Swallowing Disturbance Questionnaire (SDQ-PD) or Rastreamento de Disfagia orofaríngea em Idosos (RaDI) protocols.

Exclusion criteria were: concomitant neurological diagnoses, history of orotracheal intubation, use of an alternative feeding route or tracheostomy, history of head and neck cancer, use of Deep Brain Stimulation, limb disability or paralysis, history of chronic obstructive pulmonary disease, head and neck injuries or structural changes such as laryngectomy, advanced dementia and undergoing other speech therapy concomitantly with the research period.

### Sample size

This study, a pilot clinical trial, was designed to include a small number of participants. The sample calculation was based on data obtained from the medical records of users seen at the site, which indicated an estimated 30% improvement rate in oropharyngeal dysphagia symptoms after the intervention. The calculation used an average effect size, a significance level of α = 0.05, and a statistical power of 0.8 for unpaired group tests. Thus, it was calculated that 22 participants would be needed for the total group, with 26 being recruited to compensate for a possible drop-out rate of 20%.

### Recruitment and initial assessment

Participants were informed of all the procedures before the start of the study. The classification of the stage of the disease, recorded in the medical records by the medical team, according to the Hoehn & Yahr scale^(17)^, was taken into account. Cognitive screening was carried out using the Montreal Cognitive Assessment (MoCA)^(18)^, and the participant's level of dependence or independence in everyday tasks was assessed using the Functional Independence Measure (FIM) scale^(19)^ translated into Brazilian Portuguese by the principal investigator.

The MoCA was used as a screening tool for cognitive impairment, whose cut-off points are: ≥ 26 normal; 19-25 mild cognitive impairment; 10-18 moderate cognitive impairment; < 10 severe cognitive impairment. The FIM was used as a measure of the degree of functional dependence on another person, covering six domains of activities of daily living.

The Swallowing Disturbance Questionnaire (SDQ-PD)^(20)^, translated and adapted into Brazilian Portuguese, and the Rastreamento de Disfagia orofaríngea em Idosos (RaDI)^(21)^ were used to screen for dysphagia. Individuals with scores of 11 or more on the SDQ and 4 on the RaDI were considered eligible for the study. Confirmation of the presence of oropharyngeal dysphagia, based on the diagnostic impression, was hypothesized from the clinical assessment, carried out using the Northwestern Dysphagia Patient Check Sheet (NDPCS), a version translated and cross-culturally adapted into Brazilian Portuguese^(22)^. Oral intake was classified using the Functional Oral Intake Scale (FOIS)^(23)^, which ranges from 1 (no oral intake) to 7 (total oral intake without restrictions).

### Randomization

Simple stratified randomization was used to allocate the participants in the study. Participants were randomly assigned to two groups (A or B) using an Excel® spreadsheet. The random list generated contained numbers from 1 to 26, distributed equally between the CTAR and CD groups, so that the program could reorganize the sequence randomly. The researcher responsible for the randomization was different from the one responsible for the intervention, to ensure that the study was blind. The sequence of the groups remained hidden until a group was assigned to the participant in chronological sequence of adherence. In cases where a participant discontinued or was removed, the next volunteer who agreed to take part occupied their position in sequence.

### Interventions

Four face-to-face sessions were held, lasting an average of 40 minutes, once a week. The participants were randomly assigned to two groups. Group A received the intervention program with CTAR exercises and functional swallowing training, while Group B received the intervention program with the CD postural maneuver and functional swallowing training with different food consistencies. The liquids used for functional swallowing training in both groups were classified according to the International Dysphagia Diet Standardization Initiative (IDDSI)^(24)^. All participants underwent the face-to-face sessions while on the dopaminergic medication prescribed by the medical team.

The principal investigator attended all the face-to-face sessions to monitor the performance of the exercises. Both groups received the usual guidance on meal management, such as: (a) having meals in a quiet place without screen stimuli; (b) keeping their attention on the food; (c) avoiding having meals at times when they were sleepy or tired; (d) trying to have meals while on the medication; (e) chewing the food well before swallowing; (f) putting small portions of food in their mouths; (g) not eating in a hurry; (h) keeping their torso upright and their head held high while eating; (i) not talking while chewing.

**Figure 1:**
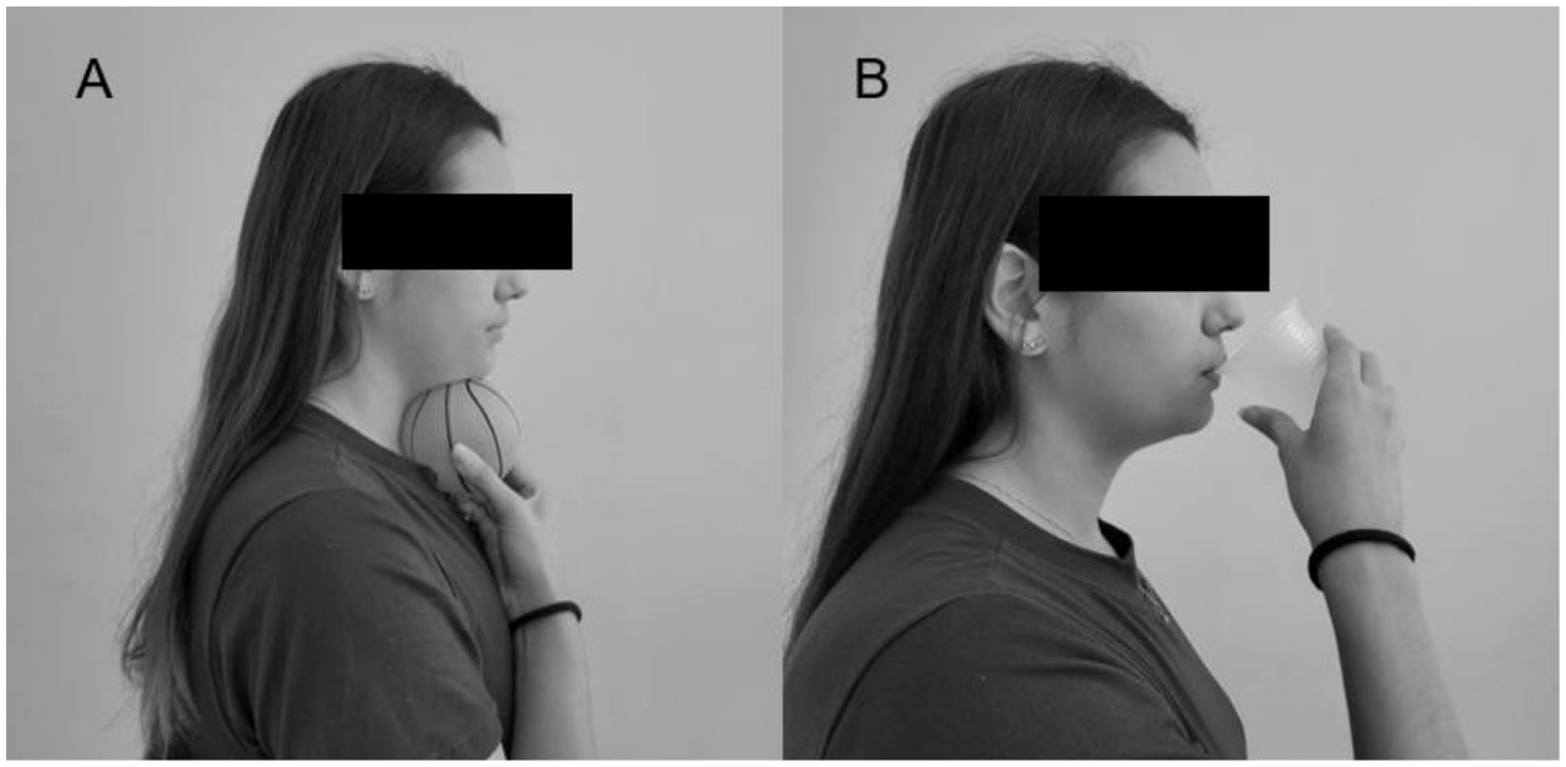
Demonstration of the intervention program with CTAR exercises. (A) In the neutral position with the ball. (B) Functional swallowing training with level 0

The individuals in Group A performed 10 sets of 10 isotonic components and 1 isometric component for 60 seconds, with a rest interval of 20 seconds between sets. To perform the CTAR exercises, an elastic ball approximately 12 cm in diameter was used, handled entirely by the participant, and positioned between the chin and the furcula of the sternum bone. The participant was instructed to remain seated, with an upright torso, feet flat on the floor, and head initially in a neutral position. They were asked to press their chin against the ball with as much voluntary force as possible, without pressing on their teeth (Figure 1).

For functional swallowing training, a glass containing 200 mL of thin liquid (level 0) according to the IDDSI classification, with no artificial flavors added, was made available throughout the face-to-face session. The participant ingested a portion of the liquid on demand after each series, totaling 200 mL at the end of the session. In addition, a cervical stretching component was performed, which consisted of rotating the head sideways for 20 seconds.

**Figure 2:**
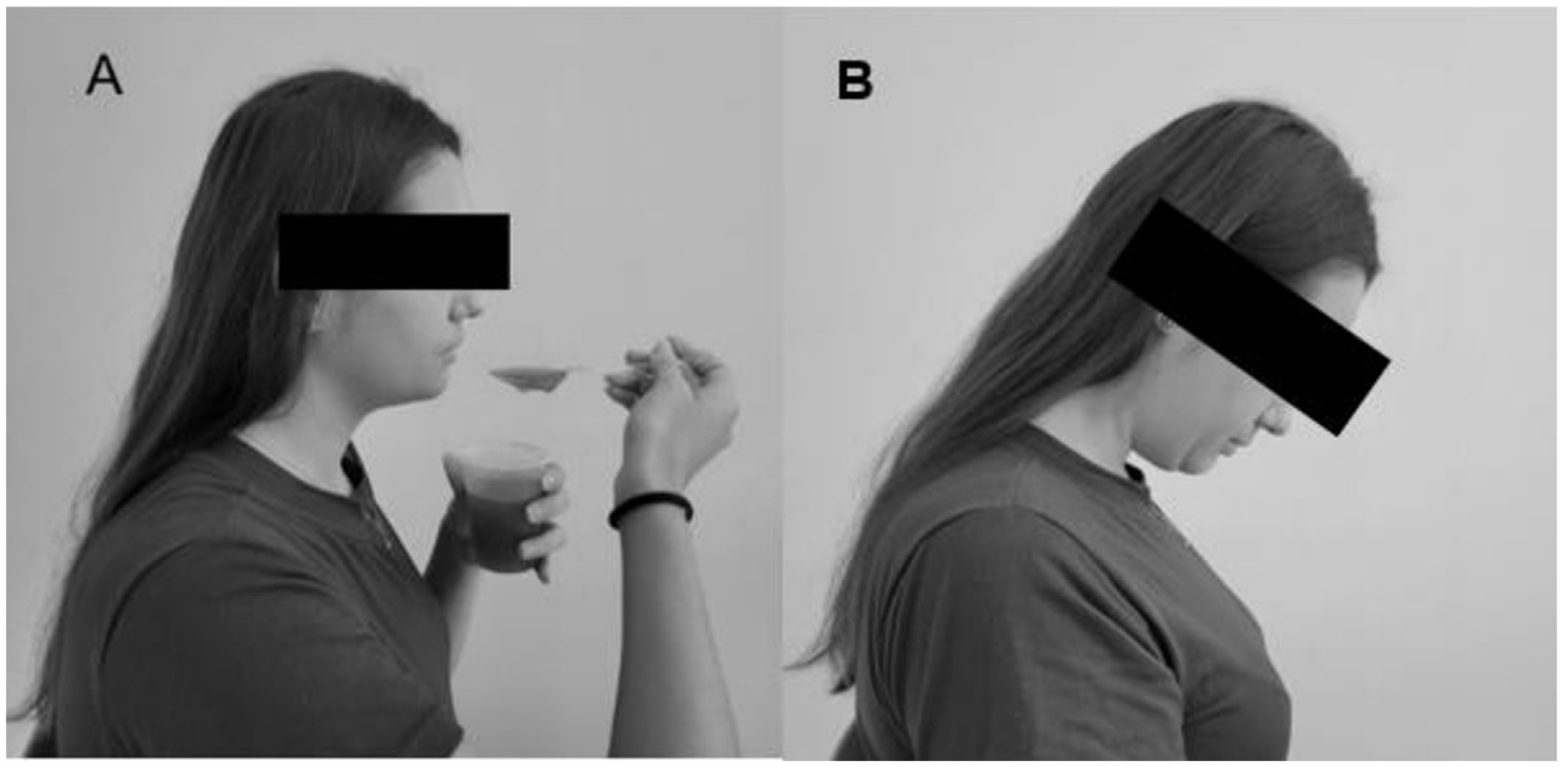
Demonstration of the intervention program with the chin-down postural maneuver. (A) Functional swallowing training with thickened liquid. (B) Chin-down postural maneuver

The individuals in Group B performed 10 repetitions of the CD postural maneuver for each food consistency. The participant was instructed to remain seated, with an upright torso, feet flat on the floor, and head initially in a vertical plane before taking the food into the oral cavity (Figure 2). Three food consistencies were used in the following order: extremely thickened liquid (level 4), slightly thickened liquid (level 2), and thin liquid (level 0) according to the IDDSI classification. The liquids were thickened with a cornstarch-based instant product, artificially flavored as grape-flavored juice, except for water, and offered in a 5 mL spoon. During the session, a cervical stretching component was performed, consisting of rotating the head sideways for 20 seconds.

It should be noted that, due to the characteristics of the CD maneuver and the viscosity of the liquids, functional training took place with a smaller volume, which is corroborated by previous studies in the literature^(12,25)^. However, in the CTAR group, as this is still an experimental intervention, there is no data available on the use of other food consistencies in functional swallowing training.

### Monitoring activities at home

During the intervention period, the participants did not receive any other speech-language therapy. In addition to the usual guidance on swallowing, they were instructed to perform the exercises of the group to which they were allocated at home, before the main meal of the day. Each participant received a printed sheet to record the days on which they performed the exercises. This sheet was checked at every face-to-face session. The team reinforced the importance of adherence to the sessions, focusing on correct execution and the right number of repetitions. Progress was monitored weekly in both groups, checking compliance with the guidelines and continuity of the protocol.

In cases where the individuals had caregivers or companions, they provided support for the activities at home, but the exercises were carried out entirely by the participants.

### Primary outcomes

The primary outcome measures were defined according to swallowing function. The assessments were carried out by experienced, independent researchers who were blinded to the interventions and the groups to which the participants were allocated. The assessments were carried out before and after the intervention period, with a maximum interval of two weeks.

The primary outcomes of the study were established according to: (a) the Northwestern Dysphagia Patient Check Sheet (NDPCS); (b) the Eating Assessment Tool (EAT-10); (c) maximum isometric tongue pressure (MIP).

The clinical assessment of swallowing was carried out by a speech therapist with previous experience in oropharyngeal dysphagia and trained to apply the NDPCS protocol translated and adapted into Brazilian Portuguese^(22)^. This is a protocol commonly used in speech therapy practice for the clinical assessment of swallowing, which, although it was not initially developed to conclude a diagnosis of oropharyngeal dysphagia, allows for an analysis of the diagnostic impression. The protocol is made up of 28 items, distributed into five categories, and at the end, it establishes a relationship between the items and the potentially altered phases of swallowing, based on the statistical inferences made in its original version^(26)^.

In the NDPCS swallowing test, different foods were offered, classified according to the IDDSI and presented in the following order: 5 mL of level 4 (extremely thick liquid), 5 and 10 mL of level 0 (thin liquid), and a single portion of level 7 wafer cookie (regular solid). Signs suggestive of penetration and aspiration were assessed and scored according to the protocol's guidelines.

The EAT-10, translated into Brazilian Portuguese^(27)^, is a protocol developed for self-assessment of swallowing difficulties and therapeutic efficacy based on the signs of dysphagia self-reported by the individual. It consists of 10 questions, scored from 0 (not a problem) to 4 (a very big problem).

MIP was measured using the Iowa Oral Performance Instrument (IOPI Medical LLC®, Redmond, WA). The portable device is used to measure the specific pressure of the tongue in kilopascals (kPa). This objective measurement was used to assess tongue muscle performance related to swallowing. The evaluator, calibrated to perform this measurement, was responsible for explaining the entire procedure to the participant. The conductive bulb was positioned in the oral cavity, on the anterior portion of the dorsum of the tongue, just behind the upper incisors. The participant was asked to press the bulb with the tongue against the hard palate, without pressing on the teeth. Three 2-second attempts were made, with a short rest interval. The highest value of the attempts was considered for analysis.

### Secondary outcome

The secondary outcome measure was defined as the possible impact of the exercises on cough function. The evaluation of the secondary outcome was carried out by other experienced researchers, independent of the evaluators of the primary outcomes, who were blinded to the intervention groups in which the participants were allocated. The secondary endpoint established was peak cough flow.

Measuring peak cough flow is an important indicator directly related to the individual's ability to protect the lower airways and expel aspirated waste. For this purpose, the MicroLife PF100®, a portable electronic device, was used to obtain the desired values. The evaluator was responsible for putting on the nose clip, holding the device, and instructing the participant to put their lips around the mouthpiece of the device and “cough as hard as possible, as if something was stopped in their throat” during the measurement of cough flow. For this variable, three attempts were made, with brief rest intervals between them. The highest value obtained was taken into account for the analysis.

### Blinding

To ensure that the study was blinded, the evaluators of the primary outcomes were different from the evaluators of the secondary outcomes, and all the evaluators were blinded to the allocation of the participants to the intervention groups and were not involved in the face-to-face intervention sessions. The principal investigator was responsible for the intervention, and due to the nature of the exercises, it was not possible to maintain blinding regarding the intervention programs carried out by the participants in each group. As only the outcome assessors were blinded, it was not necessary to disclose the groups afterwards, and no outcome assessors were involved in the data analysis. Data analysis was carried out by a third party who was not involved in the interventions or evaluations.

The data was collected by the evaluators on site, recorded on forms, and entered into the laboratory computer for later analysis of the primary and secondary outcomes. All the data obtained in the study were anonymized and recorded only by a code associated with each participant. The forms were stored in specific folders, while the data collected on the computer was organized and stored in the cloud.

### Statistical analysis

The statistical analysis was carried out using the program JAMOVI version 2.3.28. All numerical data were tested for normal distribution, and parametric tests were applied to variables that followed a normal distribution. Comparisons between the means of two groups were analyzed using the t-test for independent samples, while the association between categorical data was analyzed using Pearson's chi-squared test. Comparisons of continuous data trends over time and in different groups were investigated with an analysis of variance (ANOVA) to determine whether the groups differed in their baseline. The assumptions were checked, and a 2 x 2 (time x group) multivariate ANOVA (MANOVA) with repeated measures was applied, examining any group differences in the changes resulting from the therapeutic program between the first and second measurements. When there were changes, the Tukey test was applied to check in which group the difference occurred. The effect size was checked with Eta squared (𝜂2). Statistical significance was set at p ≤0.05 for all the analyses in the study.

## RESULTS

**Figure 3:**
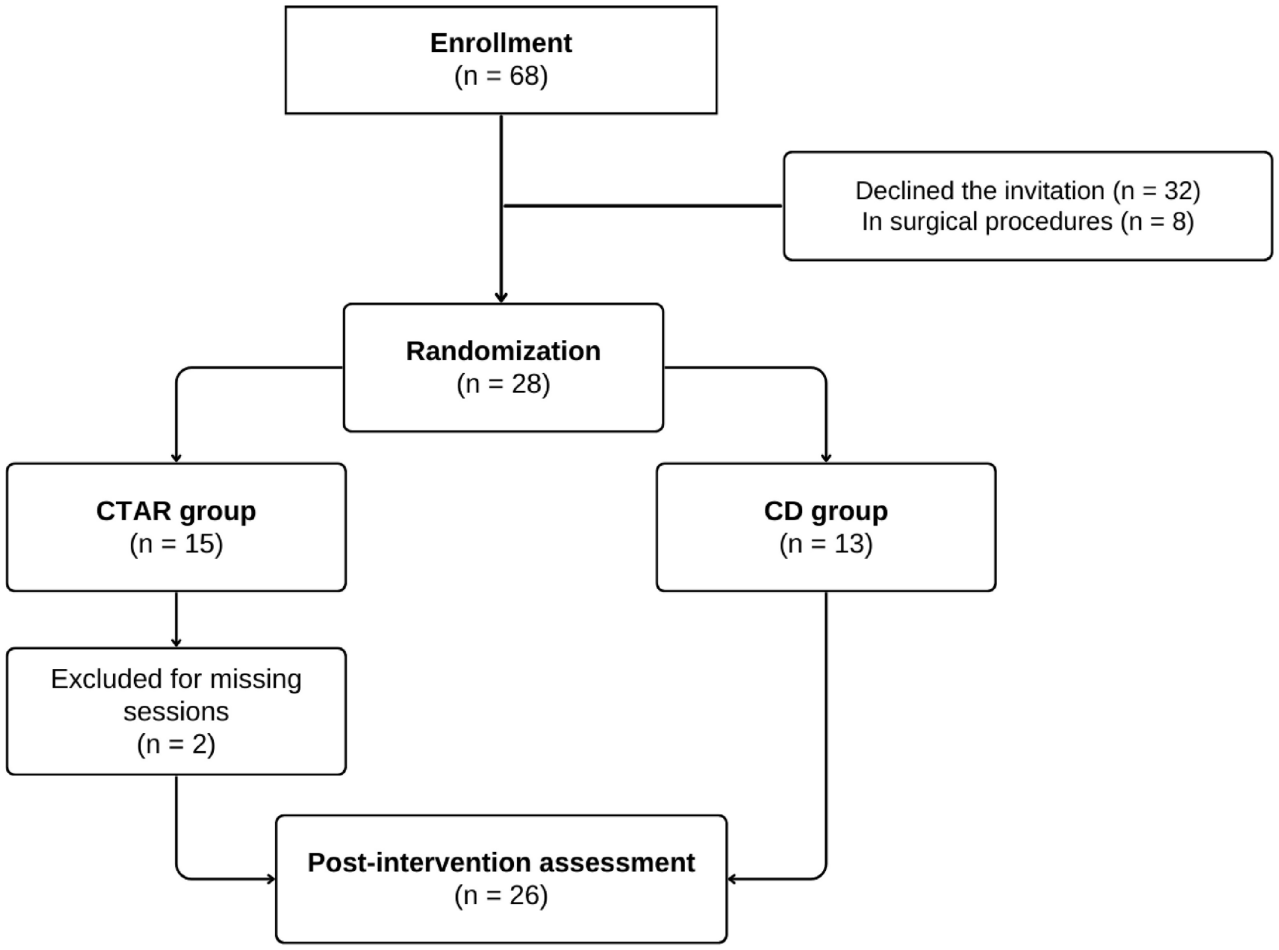
Flow chart of the participants

A total of 68 individuals with PD were recruited for the study, 42 of whom were excluded. Thus, 26 participants were analyzed and randomly assigned to two groups: 13 participants in the CTAR group and 13 in the CD group (see study flowchart, Figure 3). The sample was gender-equal, with 13 men and 13 women. The majority were elderly people over 60 (57.6%), with a minimum age of 46 and a maximum of 87. The characteristics of the participants, including age, gender, and initial assessment data, are shown in Table 1.

**Table 1:** Characteristics of the participants at the initial assessment between the groups

**Variables**	**CTAR group**	**CD group**	***p-*value**
Gender			
Male	7 (26.9)	6 (23.1)	
Female	6 (23.1)	7 (26.9)	0.695
Age (years)	60.9 ± 11.2	65.0 ± 9.2	0.320
Hoehn & Yahr stage	2.7 ± 0.9	3.0 ± 1.0	0.547
Time of diagnosis (years)	7.8 ± 3.7	8.2 ± 6.4	0.853
SDQ-PD	12.8 ± 6.6	12.0 ± 4.1	0.700
RaDI	5.6 ± 2.0	5.1 ± 2.1	0.586
FOIS	6.6 ± 0.6	6.3 ± 0.8	0.361
MoCA	21.8 ± 4.3	21.6 ± 4.5	0.896
Functional Independence Measure			
Personal Care	38.2 ± 6.1	36.1 ± 7.6	0.408
Sphincter control	11.2 ± 3.3	10.6 ± 4.3	0.956
Mobility/Transfer	20.3 ± 2.5	17.9 ± 5.5	0.082
Locomotion	11.9 ± 3.1	11.2 ± 3.8	0.648
Communication	13.7 ± 0.7	13.5 ± 0.8	0.652
Social Cognition	18.6 ± 3.9	18.5 ± 3.6	0.660
Dosage of medication (per day)	6.6 ± 2.4	5.5 ± 2.1	0.225
Dysarthia			
Yes	9 (34.6)	11 (42.3)	0.645
No	4 (15.4)	2 (7.7)	
Palilalia			
Yes	4 (15.4)	4 (15.4)	1.000
No	9 (34.6)	9 (34.6)	

The data was described as mean (standard deviation) or number (percentage).

**Caption:** CTAR = Chin Tuck Against Resistance; CD = Chin-down; SDQ = Swallowing Disturbance Questionnaire; RaDI = Rastreamento de Disfagia orofaríngea em Idosos; FOIS = Functional Oral Intake Scale; MoCA = Montreal Cognitive Assessment

There were no significant differences between the groups in terms of the participants' characteristics. The MoCA scores indicated mild cognitive impairment in the sample, while the FIM measures showed low dependence on caregivers in activities of daily living. Considering that the participants who completed the study needed to travel to their appointments, few required companions. As for the stage of the disease, the majority had mild to moderate bilateral motor impairment while maintaining functional independence.

Concerning the primary outcomes, the CTAR group showed a significant improvement in the NDPCS, MIP, and EAT-10, compared to the CD group, after the intervention period (Table 2). The effect size ranged from small to moderate among the outcomes analyzed. In the intra-group analysis, the CTAR group showed a significant improvement in all the variables analyzed before and after the intervention (p <0.001), while the CD group showed an improvement only in the EAT-10 after the intervention period (p = 0.041).

**Table 2:** Analysis of primary outcomes between groups

	**CTAR group**		***p* Tukey**	**CD group**		***p* Tukey**	**Interaction between groups**	**Effect size**
	**Before**	**After**		**Before**	**After**		***p-*value**	
NDPCS	7.5 ± 3.6	4.4 ± 3.0	**<0.001**	7.4 ± 2.0	6.9 ± 2.9	0.821	**0.008** ^*^	0.042
MIP	30.4 ± 15.6	40.9 ± 17.9	**<0.001**	35.9 ± 14.4	37.5 ± 12.9	0.867	0.006*	0.022
EAT-10	12.7 ± 4.6	4.0 ± 3.5	**<0.001**	11.2 ± 4.8	8.0 ± 5.3	**0.041**	0.002*	0.062

The data was described as mean (standard deviation)

* MANOVA

**Caption:** CTAR =Chin Tuck Against Resistance; CD = Chin-down; NDPCS = Northwestern Dysphagia Patient Check Sheet; EAT-10 = Eating Assessment Tool; MIP = Maximum Isometric Tongue Pressure

**Figure 4:**
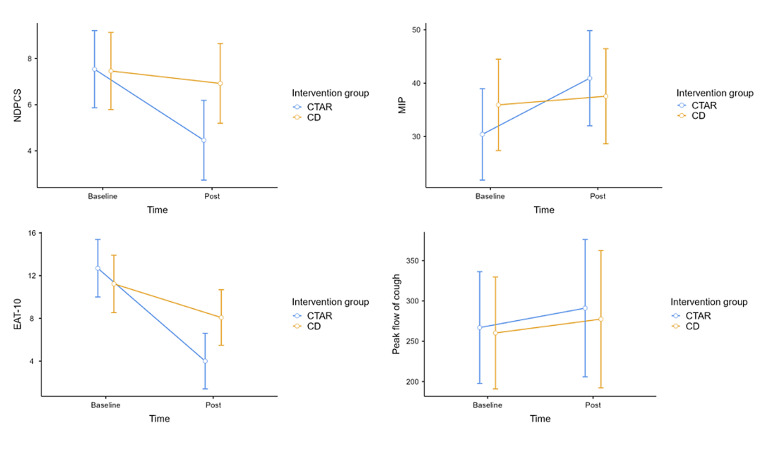
Comparison of all the outcomes analyzed between the study groups. (A) NDPCS = Northwestern Dysphagia Patient Check Sheet. (B) MIP = Maximum Isometric Tongue Pressure. (C) EAT-10 = Eating Assessment Tool. (D) Peak Flow of Cough

For the secondary outcome, there was no significant difference between the groups, nor was there any difference in the intra-group analysis for both intervention programs (Table 3). The comparison of the results for all the outcomes between the groups is shown in Figure 4.

**Table 3:** Analysis of the secondary outcome between the groups

	**CTAR group**		***p* Tukey**	**CD group**		***p* Tukey**	**Interaction between groups**
	**Before**	**After**		**Before**	**After**		***p-*value**
Peak cough flow (L/min)	267 ± 133	291 ± 145	0.826	260 ± 108	277 ± 152	0.928	0.863

The data was described as mean (standard deviation)

**Caption:** CTAR =Chin Tuck Against Resistance; CD = Chin-down

## DISCUSSION

Our data showed that individuals with PD who participated in the intervention group with CTAR exercises showed an improvement in signs suggestive of dysphagia, maximum isometric tongue pressure, and therapeutic efficacy, compared to the intervention group with the CD postural maneuver. The findings suggest that CTAR exercise therapy was effective in reducing clinical signs suggestive of penetration and aspiration.

The results found are relevant because they can benefit developing countries and regions with limited access to diagnostic health technologies, since the instruments used in the evaluation are less expensive and more accessible. The NDPCS protocol is widely used in clinical practice and has already been applied in other studies to analyze the swallowing process when there was no access to instrumental evaluation^(12,28)^. This method can provide relevant information to professionals without access to instrumental assessment of swallowing or to clinical protocols that have been validated in all stages of obtaining evidence and accuracy, such as those used in this research. However, it can be applied with the aim of aiding in the diagnostic impression of the clinical condition under study and in the analysis of therapeutic efficacy.

According to the literature, interventions with CTAR exercises are heterogeneous in terms of the number of sessions, the isotonic and isometric components, the intensity of repetitions, and the various variations of counter-resistance exercise^(15)^. For this reason, this exploratory study proposed a new method for rehabilitating swallowing function in patients with PD.

The treatment of dysphagia in PD patients aims to re-establish functional and safe swallowing, ensuring adequate nutritional status and promoting a good quality of life^(29)^. However, as reported in the literature and observed in this study, interventions for swallowing in PD tend to be more effective when applied in the early stages^(30,31)^. As symptoms progress, the decline in swallowing function often becomes irreversible. In this context, the choice of exercises to strengthen the suprahyoid muscles in the early stages of the disease brought short-term benefits for the patient, compared to the compensatory strategy.

CTAR exercises have already been shown to induce physiological changes in the target muscles in patients with early muscle weakness^(14,15,32)^, given the increase in thickness and strength of the muscle fibers subjected to the counter-resistance intensity. However, in the PD population, the physiology of movement is altered^(2)^, with the presence of dyskinesia and rigidity that hinder the isometric components of exercise. This causes their performance to fluctuate during exercise if they are not on dopaminergic drugs^(2)^.

The CD postural maneuver has already been shown to reduce signs suggestive of oropharyngeal dysphagia in the NDPCS protocol in PD^(12)^, but its effects are restricted to the immediate moment^(29)^. While CTAR exercises produced a recruitment of muscle fibers, which, combined with functional training, resulted in a greater effect on swallowing kinematics^(30)^. With the increase in the strength of contraction of the suprahyoid muscles between intensive exercise sessions, changes in the biomechanics of swallowing are expected after four weeks of the therapeutic program^(30)^.

Strengthening the suprahyoid muscles is rarely reported in the rehabilitation of PD patients^(32)^. CTAR exercises directly strengthen the suprahyoid muscles, which increases hyolaryngeal excursion during swallowing^(33)^. This, in turn, improves the lower airway protection mechanism and reduces aspiration episodes^(32)^. In this context, previous evidence using the technique has shown a reduction in aspiration episodes during the pharyngeal phase of swallowing in dysphagic patients after stroke^(34,35)^. Similarly, the present study also showed a reduction in signs suggestive of penetration and aspiration in patients with PD, demonstrating the therapeutic potential of CTAR exercises in this group.

Regarding the therapeutic program with the CD maneuver, it has a compensatory proposal in the biomechanics of swallowing, with the addition of difficult tasks in functional training. Performing this maneuver involved the process of motor learning to perform challenging tasks based on the different food consistencies used^(36)^. It is known that the more viscous consistencies require greater oral motor control for retropropulsion with head flexion^(36)^. The consolidation of this learning process takes an indeterminate amount of time, as it depends on the cortex's ability to develop new neural connections to perform the task correctly after several consecutive attempts^(37)^. Although it involves a different process from muscle strengthening, this study only found an improvement in the risk of self-reported dysphagia among participants in the CD group after the intervention period.

The motor activity of swallowing does not involve maximum muscle strength, but a coordinated sensory-motor capacity for control and mobility^(36)^. The therapeutic program based on CTAR was superior in increasing the MIP measurement compared to the postural maneuver. Anterior tongue pressure is a relevant measure for monitoring the progression of patients with dysphagia, as the suprahyoid muscles make up the floor of the oral cavity and participate in movements in the oral and pharyngeal phases of swallowing, connecting the hyoid bone with the base of the mandible and skull^(38)^. The data is in line with another study, which indicated an increase in anterior tongue pressure in healthy adults after 8 weeks of intervention, while in our study, we observed changes in four weeks^(39)^. Thus, the CTAR exercise proved to be effective in increasing anterior tongue pressure in dysphagic patients with PD, especially during the isometric components of the exercise.

Monitoring of therapeutic efficacy was one of the outcomes analyzed, and there was improvement in both intervention groups. The self-reported protocol asks about the occurrence of dysphagia symptoms in aspects of daily life and difficulties. Therefore, the reduction in everyday swallowing difficulties can also be explained by the standardized guidance on meal management given to both groups at the start of the study. On the other hand, the reduction observed only in the CD group can also be explained, in part, by the immediate action of the postural maneuver, which reduces posterior oral escape due to reduced oral control, especially with thin liquids^(12,13)^. However, the maneuver has important limitations, such as the fact that it is difficult to perform while swallowing drug capsules^(29)^.

The administration of drugs orally and the progressive difficulty that PD patients have in swallowing them with water is common^(29)^. In this context, the therapeutic program based on the postural maneuver has long-term disadvantages for ingesting liquids without thickening with the medications themselves. In our sample, we found an average of 6 doses of medication ingested per day just to treat the symptoms of the condition. Thus, using the postural maneuver with only one food consistency, water, is a limiting factor in the therapeutic process to rehabilitate the swallowing function in PD patients.

The cough parameter was assessed to verify the effects of therapeutic programs on expiratory flow, since the laryngeal muscles participate in both tasks. It is known that therapeutic programs focused on strengthening the expiratory muscles have shown positive side effects on swallowing in PD patients^(40)^. The results of the present study showed no differences between the groups after the intervention period. We believe that the principles of the therapeutic programs, such as strengthening the suprahyoid muscles and motor learning, and the intervention time, did not influence the dynamics of the subglottic pressure necessary for the coughing function.

Due to the limitations of the institution, it was not possible to carry out an instrumental assessment of swallowing, since we would have had more reliable data on the effectiveness of the therapeutic programs on the biomechanics of swallowing. However, we used other accessible assessment methods, previously used in other clinical studies [12, 25, 28], which are associated with a reduction in the signs observed in instrumental examinations.

The results of this pilot study indicated changes in the outcomes analyzed after four weeks of intervention. It is thought that a longer period, such as eight weeks, could result in more robust effects. It should be noted, however, that there may be a limit to obtaining MIP measurements due to both the natural progression of the disease and the adaptive capacity of the individuals. This hypothesis needs further investigation.

One of the limitations of this study is the variation in the stages of the disease among the participants, with a predominance of cases of mild dysphagia. This factor restricts the applicability of the findings to more severe cases. There was also a difference in intensity and number of repetitions between the two therapeutic programs. It is necessary to apply the interventions to a larger sample and include a control group for both protocols. In addition, the use of unthickened liquid in the functional training of the CTAR group could be a risk of aspiration in participants with severe dysphagia.

The EAT-10 protocol, being a self-report instrument, may be subject to bias in the subjective responses of individuals. However, the evaluator was properly trained to apply the questions in a standardized manner and was free from influences that could compromise the objectivity of the answers.

For future research, there is a need to investigate the outcomes of the instrumental assessment and include a third control group without intervention. It should be noted that the benefits of CTAR exercises in PD need to be validated through studies with greater methodological rigor, including designs with longitudinal follow-up. As a strong point, this pilot study is supposedly the first to describe the effectiveness of CTAR exercises in PD.

## CONCLUSION

Individuals with Parkinson's disease who underwent an intervention program with CTAR exercises showed an improvement in signs suggestive of dysphagia and an increase in maximum isometric tongue pressure, compared to those who underwent the therapeutic program with the chin-down postural maneuver.
